# Complete Genome Sequence of Avian Paramyxovirus Strain APMV-6/red-crested pochard/Balkhash/5842/2013 from Kazakhstan

**DOI:** 10.1128/genomeA.00158-15

**Published:** 2015-07-16

**Authors:** Kobey Karamendin, Aidyn Kydyrmanov, Aigerim Seidalina, Maria Jenckel, Elke Starick, Christian Grund, Saule Asanova, Elizaveta Khan, Klara Daulbayeva, Yermukhammet Kasymbekov, Kainar Zhumatov, Marat Sayatov, Martin Beer, Sasan Fereidouni

**Affiliations:** a Laboratory of Viral Ecology, Institute of Microbiology and Virology, Almaty, Kazakhstan; b Friedrich-Loeffler-Institut, Insel Riems, Germany; c WESCA Wildlife Network, Greifswald, Germany

## Abstract

An avian paramyxovirus 6 strain was isolated during a wild bird monitoring study in Kazakhstan in 2013. The virus was isolated from a wild duck red-crested pochard (*Netta rufina*) in southeastern Kazakhstan. Here, we present the complete genome sequence of the virus.

## GENOME ANNOUNCEMENT

Avian paramyxoviruses (APMVs) belong to the genus *Avulavirus* in the family *Paramyxoviridae* and include 12 serotypes, APMV-1 to APMV-12 (1–4). APMV-1, or Newcastle disease virus (NDV), is the most frequently isolated APMV. To date, the complete genome sequences of eight isolates of APMV-6 and partial sequences of 3 isolates are available in public databases (GenBank, updated 1 February 2015). In this study, we report the complete genome sequence of an isolate of APMV-6 in Kazakhstan determined by next-generation sequencing. This is the first report of APMV-6 from this country.

During a study in 2013 monitoring wild birds, a hemagglutinating virus was isolated from a cloacal swab sample of a red-crested pochard after inoculation into 10-day-old embryonated chicken eggs. Hemagglutination inhibition tests using reference sera specific to APMV-1 to APMV-9 revealed that the virus belongs to APMV serotype 6.

Viral RNA was extracted from infected allantoic fluid using the QIAamp viral RNA minikit (Qiagen). Pan-PMV primers (5) were used for amplification and sequencing of a short genome fragment using an ABI 3730xl DNA analyzer (Applied Biosystems). A BLAST search confirmed that the virus belongs to APMV-6, and it was designated APMV-6/red-crested pochard/Balkhash/5842/2013. The sample was negative for avian influenza viruses, as revealed by a duplex reverse transcription-PCR (RT-PCR) (6) and by a generic real-time RT-PCR (7).

For whole-genome sequencing, viral RNA was used as a template for double-stranded cDNA synthesis (Roche). The Covaris M220 ultrasonicator was used for DNA fragmentation. For library preparation, Illumina adaptors (Bioo Scientific) and SPRIworks fragment library cartridge II (Beckman Coulter) were used, with manual size selection afterward. The quality of the library was checked on a Bioanalyzer 2100 (Agilent Technologies). Quantity was determined via quantitative PCR (qPCR) with the Kapa library quantification kit (Kapa Biosystems). Paired-end sequencing was performed on an Illumina MiSeq instrument using the MiSeq reagent kit version 3. Raw sequence data were analyzed and assembled using the Genome Sequencer software suite (version 2.8; Roche).

Six out of the 8 available whole-genome sequences of APMV-6 (GenBank) contain a genome length of 16,236 nucleotides: duck/HongKong/18/199/1977, duck/Taiwan/Y1/1998, goose/FarEast/4440/2003, mallard/Belgium/12245/07, mallard/Jilin/127/2011, and mallard/Jilin/190/2011. The remaining two sequences have 16,230 nucleotides (red-necked stint/Japan/8KS0813/2008 and duck/Italy/IT4524-2/2007). The six-nucleotide (equal to amino acids QY) difference between the two groups of viruses is due to a deletion in the nontranslating end of the fusion protein gene. The studied virus belongs to the first group, with 16,236 nucleotides, and contains seven genes common to all APMV-6 viruses.

Phylogenetic studies of full-length hemagglutinin-neuraminidase (HN) genes and investigations of the antigenic cross-reactivity studies grouped the APMV-6 viruses into two separate clusters (8), the first one including the reference strain duck/HongKong/18/199/1977 and the Kazakhstan strain and the second one represented by red-necked stint/Japan/8KS0813/2008 and duck/Italy/IT4524-2/2007, both with a shortened genome. The Kazakhstani isolate showed the highest identity with mallard/Jilin/127/2011 (98.8%). This might be explained by the close geographical relation of Kazakhstan to China and potential migration of wild birds through common flyways.

### Nucleotide sequence accession number. 

The complete sequence of APMV-6/red-crested pochard/Balkhash/5842/2013 is available at GenBank under the accession no. KP762799.
